# A Facile Synthesis of Highly Functionalized 4-Arylcoumarins via Kostanecki Reactions Mediated by DBU

**DOI:** 10.3390/molecules16086313

**Published:** 2011-07-26

**Authors:** In-Taek Hwang, Sun-Ah Lee, Jin-Soo Hwang, Kee-In Lee

**Affiliations:** Green Chemistry Division, Korea Research Institute of Chemical Technology, P.O. Box 107, Yuseong, Taejon 305-600, Korea

**Keywords:** 4-arylcoumarins, Kostanecki reaction, DBU, 3,4-disubstituted coumarins, one-pot reaction

## Abstract

An efficient synthesis of 4-arylcoumarins has been accomplished via Kostanecki reactions of 2-hydroxybenzophenones with acetic anhydride employing DBU at ambient temperature. Using the same strategy, several 2-acyloxybenzophenone derivatives were readily converted to 3,4-difunctionalized coumarins. This protocol offers a notable improvement in reaction conditions for coumarin synthesis and takes advantage of its synthetic capability, especially for highly functionalized 4-arylcoumarins with structural diversity.

## 1. Introduction

The coumarin moiety represents an important class of organic heterocycles, which can be found as a key structural core in many complex natural products and biologically active compounds [1]. They are frequently associated with biological activities such as antimicrobial and anticancer, antifungal, anti-HIV, and anti-clotting [2,3,4,5], but also served as versatile precursors of many organic transformations in the synthesis of a number of drug-like molecules [6,7]. Moreover, coumarin-based dyes and pigments are organic fluorescent materials exhibiting unique photochemical and photophysical properties, which render them useful in a variety of applications such as dye lasers, anion sensors, organic light emitting diodes, and solar cells [8,9]. These growing needs have stimulated interest in the design and efficient synthesis of highly functionalized coumarins.

So far, a number of coumarin derivatives have been synthesized by classical transformations using Pechmann, Perkin, Knoevenagel, and Reformatsky reactions as the key synthetic steps. One of the most widely used methods for the synthesis of 4-substituted coumarins is the Pechmann reaction which involves the condensation of phenols with β-ketoesters in the presence of protic or Lewis acids [10]. Recently, the Pechmann reaction has been carried out successfully using heterogeneous catalysts under microwave irradiation or in ionic liquids [11,12,13]. However, our initial attempts to prepare non-phenolic 4-arylcoumarins using the Pechmann conditions proved to be problematic, as yields around 20%–30% were achieved. Obviously, this protocol has been applied to activated phenols, mostly containing electron-donating groups at the *m*-position. Conventionally, 4-arylcoumarins have been obtained from 4-tosyloxycoumarin or its equivalent with a variety of arylating agent under transition metal-catalyzed coupling conditions [14,15,16]. A few other methods involving a domino Heck reaction/cyclization process between cinnamates with aryl halides [17], Cu-catalyzed hydroarylation of phenylpropiolates with arylboronic acids [18], and Wittig-type olefination of *2*-hydroxybenzophenone with ethyl (triphenylphosphoranylidene)acetate have been reported [19]. In this respect, the transition metal-catalyzed cross couplings are the predominantly employed strategies for the synthesis of 4-arylcoumarins.

As part of coumarin diversity-oriented synthesis, we needed a practical synthesis of highly functionalized 4-arylcoumarins without the use of expensive chemicals and noble metals. Herein we report a facile route to highly substituted 4-arylcoumarins via the Kostanecki condensation of 2-hydroxybenzophenones with acid anhydrides or acyl halides employing DBU.

## 2. Results and Discussion

The Kostanecki reaction involves the formation of coumarins or chromones from readily available 2-hydroxyarylketones and an aliphatic anhydride in the presence of the corresponding sodium salt via *O*-acylation and aldol condensation. An approach for the synthesis of 4-arylcoumarins via the Kostanecki reaction has already reported, however, that it suffers from limitations including the use of a large excess of reagents and harsh reaction conditions resulting in low yields [20]. Indeed, 4-arylcoumarin **3a** was prepared from 2-hydroxybenzophenone **1a** and excess acetic anhydride as the solvent and reagent with sodium acetate by heating for 72 h at 190 °C (Table 1, entry 2). The choice of base is of great importance in such aldol condensations; however, it has been sparsely investigated to date [21,22,23]. Thus, we undertook a survey of organic bases to examine the feasibility of the Kostanecki reaction of **1a**. The initial attempt with piperidine was useless and only prone to liberate the acetate **2a** (entry 3). It is interesting to note that coumarin derivatives have been prepared by Knoevenagel condensation of 2-hydroxybenzaldehyde and benzophenone with active methylene compounds in the presence of piperidine [21] and DBU [23], respectively. After the investigation of various bases for the ring annulations, we found amidine bases were effective and DBU in acetonitrile was the best choice for the synthesis of **3a** [14,16] affording a 86% yield at 90 °C and 82% yield at ambient temperature, respectively, only after employing 3 equiv. of acetic anhydride (entries 6 and 7). There was no significant difference in yield regarding the reaction temperature. Using the optimized conditions, we next examined the substrate scope for this reaction. The other 2-hydroxybenzophenone derivatives **1b–1d** were readily prepared in 81%**–**95% yields through the Fries rearrangement of the corresponding phenyl benzoates in the presence of aluminum chloride according to the known procedures [24,25]. It was found that the substrates tested were uniformly converted to the corresponding 4-arylcoumarin derivatives (entries 8**–**10). The spectral data of **3b** and **3d** were in agreement with those reported in the literature [14]. This result suggests that utilization of *o*-hydroxybenzophenones as acyclic precursors would be an attractive route to access this type of molecules, with respect to ready availability and easy manipulation.

**Table 1:** Kostanecki acylation of 2-hydroxybenzophenones with acetic anhydride. 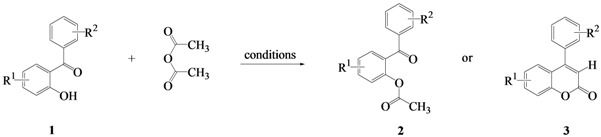

Entry ^a,b^	1	R^1^	R^2^	Conditions	2 or 3 (% yield) ^c^	Lit.
1	**1a**	H	H	KOAc, 150 °C, 4 h	**2a** (94)	
2				KOAc, 190 °C, 72 h	**3a** (71)	
3				piperidine, 150 °C, 72 h	**2a** (95)	
4				DABCO, MeCN, reflux, 20 h	**3a** (17) ^d^	
5				DBN, MeCN, reflux, 20 h	**3a** (48) ^d^	
6				DBU, MeCN, reflux, 8 h	**3a** (86)	
7				DBU, MeCN, rt, 20 h	**3a** (82)	[14,16]
8	**1b**	5-CH_3_	H	DBU, MeCN, rt, 10 h	**3b** (89)	[14]
9	**1c**	5-F	4’-Cl	DBU, MeCN, rt, 30 h	**3c** (82)	-
10	**1d**	4,6-(CH_3_)_2_	4’-CH_3_	DBU, MeCN, rt, 20 h	**3d** (53)	[14]

^a^ For entries 1–3, excess acetic anhydride was used both as solvent and reagent; ^b^ For entries 4–10, 2 equiv. of acetic anhydride and 3 equiv. of organic base were used; ^c^ Isolated yield (%); ^d^ The reaction was not completed at the indicated time.

We then evaluated the same strategy using several 2-acyloxybenzophenone derivatives, aiming to synthesize 3,4-difunctionalized coumarins. It is interesting to note that the synthesis of 3,4-difunctionalized coumarins has rarely been investigated up to date, except for few examples [26,27]. They frequently require expensive catalysts, laborious multi-step procedures, and long reaction times. The substrates, 2-acyloxyaryl derivatives **4a–4l**, could be easily prepared in 84%–94% yields by the treatment of the corresponding 2-hydroxyarylketones and acid anhydrides or acyl chlorides with Et_3_N and a catalytic amount of DMAP in chloroform at ambient temperature for 2 h. Indeed, we achieved a very convenient synthesis of 3-ethyl-4-phenylcoumarin **5a** in 47% yield by simply mixing **4a** using 2 equiv. of DBU in acetonitrile (Table 2, entry 1). Several 3,4-difunctionalized coumarins shown in Table 2 were successfully synthesized employing the established DBU-mediated reaction of 2-acyloxybenzophenones. Although the yields were moderate, a range of substrates containing electronically and sterically demanding groups on both the aryl rings proved to be compatible with this operation.

**Table 2:** DBU-mediated synthesis for 3,4-difunctionalized coumarins ^a^.

Entry	4		Time (h)	5		Yield (%)
1	**4a**	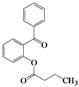	72	**5a**	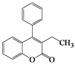	47
2	**4b**	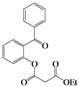	72	**5b^b^**	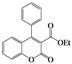	38
3	**4c**	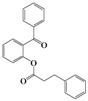	72	**5c**	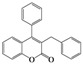	48
4	**4d**	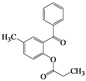	20	**5d**	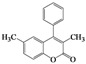	43
5	**4e**	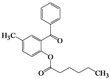	40	**5e**	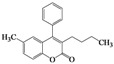	39
6	**4f**	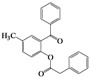	2	**5f**	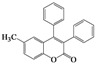	60
7	**4g**	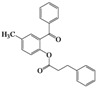	20	**5g**	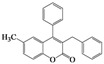	48
8	**4h**	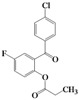	3	**5h**	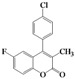	40
9	**4i**	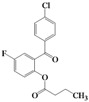	5	**5i**	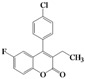	44
10	**4j**	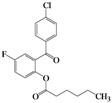	6	**5j**	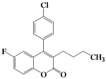	35
11	**4k**	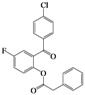	2	**5k**	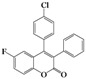	50
12	**4l**	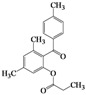	20	**5l**	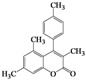	42

^a^ All reactions were carried out in acetonitrile using 2 equiv. of DBU at room temperature; ^b^ Known compound [23].

It is noteworthy that the substrates having an electron-withdrawing group such as fluorine smoothly provided the corresponding 4-arylcoumarins, which were previously difficult to obtain using the Pechmann protocol. Thus we provided a facile route to 3,4-difunctionalized coumarins via the intermolecular cyclization of 2-acyloxybenzophenone derivatives employing DBU. It appears that this methodology could be quite useful for the synthesis of highly functionalized 4-arylcoumarin materials with structural diversity.

**Scheme 1:**
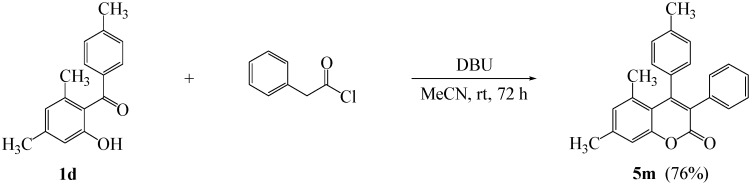
One-pot preparation of 3,4-diarylcoumarin **5m**.

Further, we thought that a one-pot preparation avoiding isolation of acyl intermediates would improve the utility of this method. Indeed, we observed the one-pot preparation of 3,4-diarylcoumarin **5m** in 76% yield, when 2-hydroxybenzophenone **1d** and phenylacetyl chloride were simply reacted in the presence of DBU, as shown in Scheme 1.

## 3. Experimental

### 3.1. General

All of the chemicals used in this work were of analytical grade purchased from commercial suppliers and used without further purification. The reactions were monitored by TLC with Merck silica gel 60 F_254_ TLC glass plates and the products were purified by flash chromatography with Merck Kiegel 60 silica gel (particles size 0.040**–**0.063 mm) using a glass column. Melting points were measured on a Clarkson IA9200 Electrothermal Melting Point apparatus and are uncorrected. ^1^H- and ^13^C-NMR spectra were recorded at 300 MHz and 75 MHz respectively on a Jeol Eclipse FT 300 MHz Spectrometer in CDCl_3_ as solvent using TMS as internal standard and chemical shifts are expressed as δ ppm. Mass spectra were detected by electronic impact (EI) and obtained on an Agilent 1100 Series VLL or JEOL the MStation JMS 700 mass Spectrometer.

### 3.2. Typical Procedure for the Synthesis of 4-Phenyl-2*H*-chromen-2-one *(**3a**)*

To a mixture of 2-hydroxybenzophenone **1a** (300 mg, 1.51 mmol) and acetic anhydride (0.28 mL, 3.02 mmol) in acetonitrile (5 mL) was added DBU (0.67 mL, 4.54 mmol) and the reaction mixture was allowed to stir for 8 h at room temperature. The solvent was removed *in vacuo* and the residue was taken up with ethyl acetate (10 mL). The organic layer was washed with water, dried over anhydrous sodium sulfate, and then concentrated to dryness. The crude was purified by column chromatography on silica gel (hexane/EtOAc = 10/1) to afford 289 mg (86% yield) of **3a** as a solid: mp 78–80 °C; Lit. 74–76 °C [16]; ^1^H-NMR (CDCl_3_, 300 MHz) δ 7.54 (m, 4H), 7.44 (m, 4H), 7.23 (m, 1H), 6.37 (s, 1H); ^13^C-NMR (CDCl_3_, 125 MHz) δ 160.7, 155.6, 154.2, 135.2, 131.9, 129.70, 128.8, 128.4, 127.0, 124.1, 118.9, 117.3, 115.1; EIMS (70 eV) *m/z* (rel intensity) 222 (M^+^, 85), 194 (95), 165 (100), 138 (30), 62 (63).

### 3.3. Typical Procedure for the Synthesis of 3,6-Dimethyl-4-phenyl-2*H*-chromen-2-one *(**5d**)*

To a solution of **4d** (194 mg, 0.72 mmol) in acetonitrile (5 mL) was added DBU (216 μL, 1.446 mmol) and the reaction mixture was allowed to stir for 20 h at room temperature. The solvent was removed *in vacuo* and the residue was taken up with ethyl acetate (10 mL). The organic layer was washed with water, dried over anhydrous sodium sulfate, and then concentrated to dryness. The crude was purified by column chromatography on silica gel (hexane/EtOAc = 25/1) to afford 78 mg (43% yield) of **5d** as an oil; ^1^H-NMR (CDCl_3_, 300 MHz) δ 7.57–7.49 (m, 3H), 7.26–7.22 (m, 4H), 6.76 (s, 1H), 2.26 (s, 3H), 1.98 (s, 3H); ^13^C-NMR (CDCl_3_, 75 MHz) δ 162.5, 150.6, 150.5, 135.0, 133.5, 131.4, 128.8, 128.5, 128.2, 126.6, 122.7, 120.3, 116.2, 20.8, 14.7; EIMS (70 eV) *m/z* (rel intensity) 250 (M^+^, 90), 221 (52), 178 (85), 165 (30), 152 (31), 115 (61).

### 3.4. Typical Procedure for the Synthesis of 5,7-Dimethyl-3-phenyl-4-(4’-methylphenyl)-2*H*-chromen-2-one *(**5m**)*

To a mixture of **1d** (80 mg, 0.33 mmol) and phenylacetyl chloride (88 μL, 0.67 mmol) in acetonitrile (3 mL) was added DBU (150 μL, 1.0 mmol) and the reaction mixture was allowed to stir for 72 h at room temperature. The solvent was removed *in vacuo* and the residue was taken up with ethyl acetate (10 mL). The organic layer was washed with water, dried over anhydrous sodium sulfate, and then concentrated to dryness. The crude was purified by column chromatography on silica gel (CHCl_3_/EtOAc = 60/1) to afford 85 mg (76% yield) of **5m** as a solid: mp 200–202 °C; ^1^H-NMR (CDCl_3_, 300 MHz) δ 7.18–7.11 (m, 4H), 7.03–6.99 (m, 4H), 6.91 (d, 2H, *J* = 8.1 Hz), 6.81 (s, 1H), 2.39 (s, 3H), 2.27 (s, 3H), 1.68 (s, 3H); ^13^C-NMR (CDCl_3_, 75 MHz) δ 161.1, 154.2, 152.7, 141.6, 137.7, 137.6, 134.9, 134.3, 130.3, 129.8, 128.6, 127.5, 127.2, 127.0, 116.3, 115.5, 23.1, 21.2; EIMS (70 eV) *m/z* (rel intensity) 340 (M^+^, 95), 325 (38), 312 (100), 297 (17), 252 (11), 189 (10), 126 (9), 105 (12).

## 4. Conclusions

In summary, we have developed a simple and efficient synthesis of highly functionalized 4-aryl-coumarins from 2-hydroxybenzophenones and their acyl derivatives via Kostanecki reaction using DBU as base. This process leads a notable improvement in reaction conditions for coumarin synthesis by Kostanecki condensation and takes advantage of its capability in the synthesis especially of highly functionalized 4-arylcoumarins with structural diversity. Moreover, a one-pot preparation of 3,4-difunctionalized coumarins has been achieved employing the same protocol. Further investigation regarding to scope of one-pot synthesis of highly functionalized 4-arylcoumarin materials is in progress.
